# Anti-HBc: a significant host predictor of spontaneous HBsAg seroclearance in chronic hepatitis B patients - a retrospective longitudinal study

**DOI:** 10.1186/s12876-023-02983-1

**Published:** 2023-10-06

**Authors:** Karin Kan, Danny Ka-Ho Wong, Rex Wan-Hin Hui, Wai Kay Seto, Man-Fung Yuen, Lung-Yi Mak

**Affiliations:** 1 Department of Medicine, School of Clinical Medicine, The University of Hong Kong, Hong Kong, China; 2 State Key Laboratory of Liver Research, The University of Hong Kong, Hong Kong, China

**Keywords:** Hepatitis B Virus, Hepatitis B Surface Antigen, Seroclearance, Hepatitis B core antigen, Anti-Hepatitis B core total antibodies

## Abstract

**Background and Aim:**

: In chronic hepatitis B infection (CHB), seroclearance of hepatitis B surface antigen (HBsAg) is associated with favourable clinical outcomes compared to those with persistent HBsAg seropositivity, and thus considered as a desired treatment endpoint. This current study explores the possibility of serum antibody to hepatitis B core antigen (anti-HBc) as a potential predictive factor of HBsAg seroclearance.

**Methods:**

This is a retrospective study that analyzed the plasma samples of CHB patients using the LUMIPULSE® G1200 analyzer. The longitudinal anti-HBc level between patients who subsequently achieved HBsAg seroclearance (S-losers) and those with persistent HBsAg-positivity (controls) were compared at multiple time points before the event.

**Results:**

A total of 240 subjects (120 S-losers and 120 controls; age- and gender-matched) were included (mean age 56.42 ± 10.81, 65% male). Compared to controls, S-losers had significantly lower plasma anti-HBc levels prior to HBsAg seroclearance, with a significant trend of declining plasma anti-HBc 8–5 years prior to HBsAg seroclearance (p < 0.01), while such trend was not observed in controls. ROC curve analysis revealed that plasma anti-HBc at multiple time points before HBsAg seroclearance return AUC greater than 0.7. Plasma anti-HBc level at the cut-off value of 82.50 COI was 68.3% sensitive and 90% specific for HBsAg seroclearance within 1 year. Combining with quantitative HBsAg < 100 IU/mL, anti-HBc < 82.5 COI identified 88.2% patients who would develop HBsAg seroclearance within 1 year.

**Conclusion:**

Plasma anti-HBc level began to decline 10 years prior to HBsAg seroclearance and can serve as a potential predictor for subsequent HBsAg seroclearance.

## Introduction

Hepatitis B virus (HBV) is a hepatotropic virus that can cause a range of clinical conditions in humans. The severity of HBV infection can vary, resulting in either acute or chronic hepatitis B infection (CHB). The risk of chronicity is largely determined by the host’s immune system response and how the virus interacts with the host’s body. The earlier in life an individual is infected with the virus, the higher the likelihood of developing chronic infection [1]. According to data from 2019, there are approximately 1.5 million new cases of chronic hepatitis B each year and 296 million people, or 4% of the world’s population, are living with chronic hepatitis B [2]. In the same year, hepatitis B was accountable for 523,000 deaths globally due to cirrhosis and hepatocellular carcinoma (HCC) [2]. Seroclearance of hepatitis B surface antigen (HBsAg) is a rare event that occurs at an annual rate of 1% in which patients with CHB developed seroclearance of HBsAg [3]. Spontaneous HBsAg seroclearance is defined as, not under any interference of drugs, being tested negative for HBsAg on 2 occasions that are at least 6 months apart, with or without seroconversion to antibody to HBsAg (anti-HBs) [4]. CHB patients that achieve HBsAg seroclearance have favourable clinical outcomes compared to those with persistent HBsAg seropositivity. HBsAg seroclearance invariably accompanies substantial decreases in all HBV-related markers or symptoms, such as HBV DNA and inflammation of livers, reflecting attenuation of HBV activity [5]. Several longitudinal follow-up studies show that patients with HBsAg seroclearance without evidence of cirrhosis or co-infection have a lower risk of cirrhosis and HCC development compared to those who remained seropositive for HBsAg [6–10]. In Fattovich’s study, 3% of patients with HBsAg seroclearance develop HCC versus 11% in patients that remain HBsAg-positive [11], whereas in Arase’s study, none of the non-cirrhotic patients with HBsAg seroclearance developed HCC [12]. Reversal of liver fibrosis is also seen in 70% of patients with HBsAg seroclearance versus 30% of patients with persistent seropositivity for HBsAg [13].

As HBsAg seroclearance is associated with a good prognosis in CHB, it is currently recognised as the desirable treatment endpoint, also known as functional cure, and has thus attracted great clinical and research interest [14]. For years researchers have been looking for factors that predict or are associated with functional cure of CHB. Viral factors that has been demonstrated to correlate with the incidence of HBsAg seroclearance include HBeAg(-) [15], low baseline HBsAg and HBV DNA levels [15, 16]. Host factors that has been demonstrated to be associated with HBsAg seroclearance include age, male [15, 17–22], hepatic steatosis [23–26], several single nucleotide polymorphisms (SNPs) [27–29], or host epigenetics regulatory elements like microRNA [30]. Another biomarker, antibody to hepatitis B core antigen (anti-HBc), is detectable in the blood among people with history of exposure to HBV. In contrast to anti-HBs – a neutralizing antibody which can form complexes with HBsAg to control HBV spread [31], the function of anti-HBc is largely unknown apart from being a marker of viral exposure [32]. A study carried out in Taiwan found that CHB patients with anti-HBc levels < 3 log IU/mL with undetectable HBV DNA had significantly greater odds in achieving HBsAg seroclearance within 10 years [33]. Another study found that low baseline anti-HBc IgG levels were associated with HBsAg seroclearance in patients who underwent nucleoside analogue (NUC)-induced HBeAg seroclearance [34, 35].

The current study is designed to explore the possibility of quantifying anti-HBc, a non-neutralizing antibody generated by host immune system in response to chronic hepatitis B infection, as a potential predictive marker of subsequent spontaneous HBsAg seroclearance.

## Materials and methods

### Study population and format

This study is a retrospective, longitudinal case-control study that used data and plasma samples collected from 2005 to 2021 from the liver clinic at Queen Mary Hospital in Hong Kong. During this period, a total of 360 chronic hepatitis B (CHB) patients developed HBsAg seroclearance. After exclusion of subjects that did not have retrievable plasma samples or did not fulfil the criteria listed below, the study eventually included 120 patients with CHB who developed spontaneous HBsAg seroclearance (referred to as “s-losers”) and age- and gender-matched with 120 treatment-naïve patients with CHB who remained seropositive for HBsAg (referred to as “controls”) in a 1:1 ratio. Plasma levels of anti-HBc were measured at various time points up to 10 years before HBsAg seroclearance or the reference point for the control group. The time points used were 10–8 (-10 to -8), 8–5 (-8 to -5), 5–3 (-5 to -3), 3–1 (-3 to -1), and 1–0 (-1 to 0) years prior to HBsAg seroclearance. All participating CHB patients were negative for HBeAg. Patients with cirrhosis, HCC, other chronic liver diseases, co-infections with hepatitis C virus, hepatitis D virus and human immunodeficiency virus were excluded. The study follows the Declaration of Helsinki. This study received approval from The Institutional Review Board of the University of Hong Kong/ Hospital Authority Hong Kong West Cluster (HKU/HA HKW IRB). The IRB reference number is UW 20–426. All participants provided written informed consent.

### Plasma anti-HBc concentration quantification via CLEIA

The plasma levels of anti-HBc IgG in 120 S-losers and 120 controls were measured using the Lumipulse® G HBcAb-N Immunoreaction cartridges set (Fujirebio, Tokyo, Japan) in a two-step sandwich chemiluminescent enzyme immunoassays (CLEIA) system working on a fully automated LUMIPULSE® G1200 analyzer (Fujirebio, Tokyo, Japan). Anti-HBc IgG levels were reported as cut-off index (COI), calculated as a multiple of the cut-off value obtained from calibration data (COI = S/C × 0.09). According to the manufacturer, the lower limit of the quantitative assay (LLOQ) is 1 COI.

### Plasma HBV DNA quantification

HBV DNA in patients’ plasmas was quantified by the Abbott HBV real-time PCR (samples collected from 2005 to 2011) or Roche Cobas HBV Test (samples collected from 2011 to 2021). HBV DNA levels were reported as IU/mL (1 IU/mL = 3.41 copies for Abbott HBV real-time PCR; 1 IU/mL = 5.26 copies for Roche Cobas HBV Test). The lower limit of both quantitative assays (LLOQ) are 10 IU/ml. The values below LLOQ were arbitrarily taken as 10 IU/mL.

### Serum HBsAg quantification

Serum quantitative HBsAg (qHBsAg) was measured by the Cobas Taqman assay (Roche Diagnostics, Gmbh, Mannheim, Germany) with a lower limit of detection (LLOD) of 0.05 IU/mL. Values were log transformed, and those values below LLOD were arbitrarily taken as 0.05 IU/mL.

### Statistical analyses

Continuous variables were expressed as mean ± standard error of mean (SEM). Variables of the s-loser and control groups at a given time point were compared using the independent student’s t-test. Significances of intra-group changes in continuous variables over time were determined using the repeated measures ANOVA, whereas McNemar tests are used for categorical variables. Receiver operating characteristic (ROC) analysis was used to determine the cut-off value for any markers that show potential predictive utility for HBsAg seroclearance in CHB patients. An area under ROC (AUC) ≥0.7 is considered to be an acceptable predictive model. The cut-off value determination is a trade-off between clinical sensitivity and specificity. Here, the cut-off value is optimized to yield at least 70.0 sensitivity and specificity. If that is not possible, specificity is prioritized over sensitivity to avoid false positives in predicting HBsAg seroclearance. All statistical analyses were performed by IBM SPSS Statistics 28 (IBM, Armonk, NY, USA). Statistical significance was defined by a p-value of less than 0.05.

## Result

A total of 240 subjects (120 HBsAg-losers and 120 controls) were included (median age 56.42 ± 10.81 years old, 65% male). The age, gender and HBV DNA at 10–8 years prior to HBsAg seroclearance or matched index time point were balanced between the two groups (Table 1). Notably, the baseline qHBsAg levels were already significantly lower in s-losers compared to controls at 10–8 years prior to HBsAg seroclearance (1.57 vs. 2.76 log IU/mL; p < 0.001). The HBV DNA levels and qHBsAg at the time of HBsAg seroclearance in s-losers were significantly lower than that in controls (p = 0.01 & p < 0.001, respectively); Table 1.

**Table 1:** Clinical characteristics of the populations quantified for plasma anti-HBc level. *Baseline refers to the serum levels of biomarker at 10–8 years before HBsAg seroclearance (reference point for controls). The HBV DNA levels and qHBsAg at the time of HBsAg seroclearance in s-losers were significantly lower than that in controls

Characteristics	S-loser group (n = 120)	Control group (n = 120)	P-value
Sex (M/F)	65/55	65/55	1.00
Age (Years)	56.45 ± 11.45	56.86 ± 11.32	0.81
*Baseline HBV DNA (log_10_ IU/mL)	4.79 ± 4.55	6.91 ± 6.70	0.32
*Baseline qHBsAg (log_10_ IU/mL)	1.57 ± 1.19	2.76 ± 0.94	< 0.001
HBV DNA at year 0 (log IU/mL)	2.64 ± 2.57	6.74 ± 6.67	0.01
qHBsAg at year 0 (log_10_ IU/mL)	-1.15 ± 0.31	2.16 ± 1.03	< 0.001

### Comparison of plasma anti-HBc levels at each timepoint

**Fig. 1:**
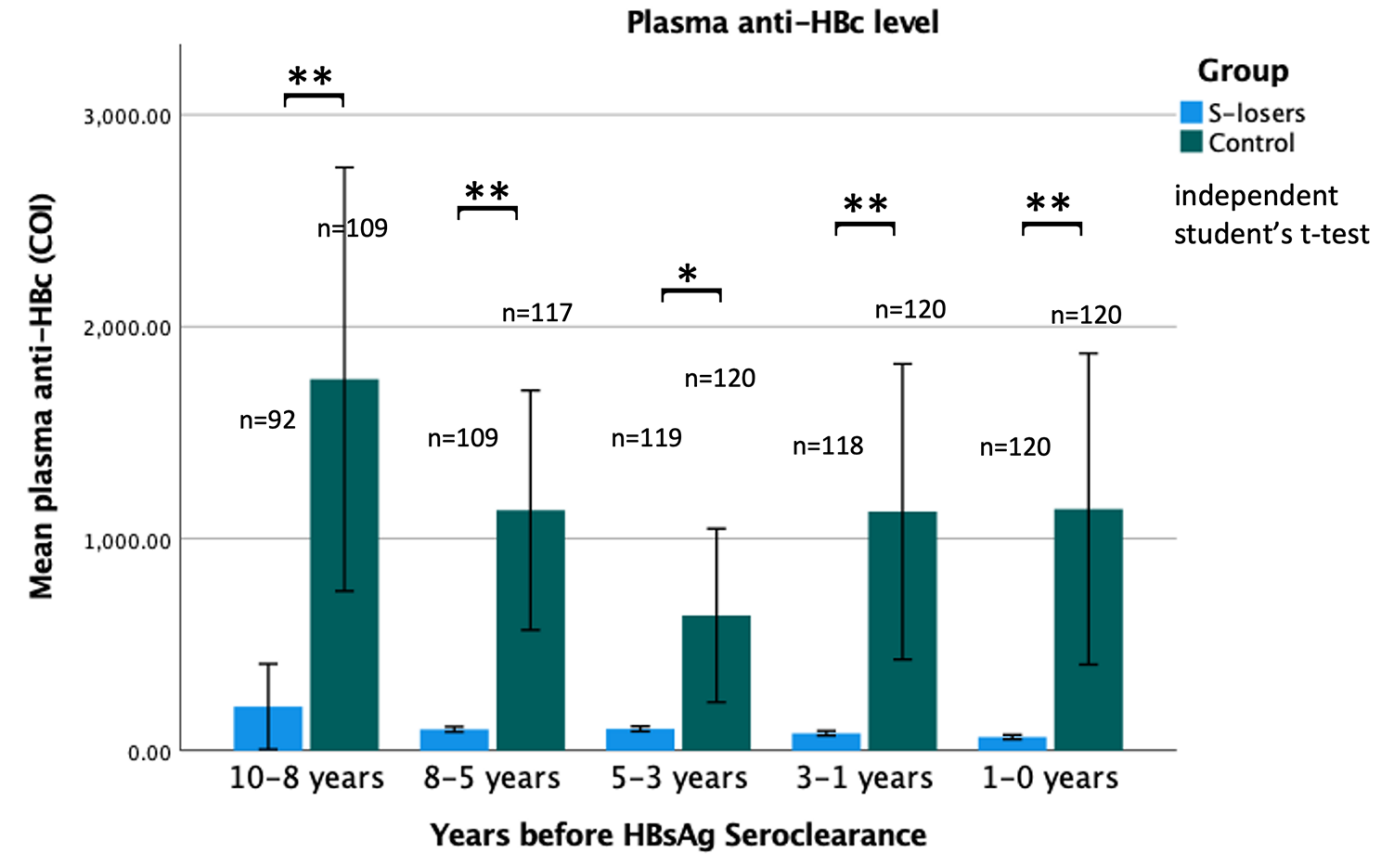
**Plasma anti-HBc levels in S-losers and controls at various time points.** Data are represented as the mean values from the sample size of a given time point of a particular group. Error bars represent the 95% confidence interval (CI). Data reaching statistically significance at p < 0.05 level are denoted by a single asterisk and Data reaching statistically significance at p < 0.01 level are denoted by double asterisks

A significant difference in plasma anti-HBc level can be observed between S-losers and controls. As shown in Fig. 1, the baseline plasma anti-HBc levels in s-losers were significantly lower than that in controls at 10–8 years (p = 0.003), 8–5 years (p < 0.001), 5–3 years (p = 0.011), 3–1 years (p = 0.004), 1–0 years (p = 0.004) before HBsAg seroclearance.

### Dynamic change in plasma anti-HBc levels over time

**Fig. 2:**
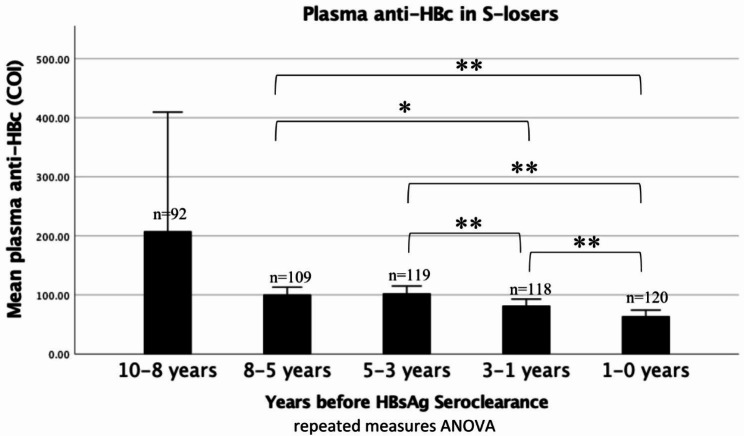
**The plasma anti-HBc levels in S-losers over the 10 years before HBsAg seroclearance.** Data are represented as the mean values from the sample size of a given time point. Error bars represent the 95% confidence interval (CI). Data reaching statistically significance at p < 0.05 level are denoted by a single asterisk and Data reaching statistically significance at p < 0.01 level are denoted by double asterisks

Significant declines in plasma anti-HBc levels over time within the S-loser group were observed. As shown in Fig. 2, the plasma anti-HBc levels in S-losers at -10 to -8 years and − 8 to -5 years are 207.92 ± 101.54 and 100.59 ± 6.29 COI respectively. Plasma anti-HBc levels further declined significantly from 102.66 ± 6.25 COI since 5–3 years before HBsAg seroclearance to 82.03 ± 5.54 COI at -3 to -1 years and 64.07 ± 5.14 COI at -1 to -0 years, with declines between each consecutive time point all reaching statistical significance (p < 0.001). Meanwhile, no significant changes were detected within the control group, with the mean plasma anti-HBc level at -10 to -8 years, -8 to -5 years, -5 to -3 years, -3 to -1 years, and − 1 to 0 years being 1752.34 ± 504.26, 1134.49 ± 285.36, 637.88 ± 206.87, 1127.35 ± 352.17, and 1140.22 ± 370.63 COI, respectively (p > 0.05 for all subgroup comparisons and overall trend). The mean decline in anti-HBc levels from 10 to 8 years to -1–0 years was not significantly different between s-losers and controls (142.52 vs. 574.37 COI; p = 0.490).

### Performance characteristics for plasma anti-HBc to predict subsequent HBsAg seroclearance

**Fig. 3:**
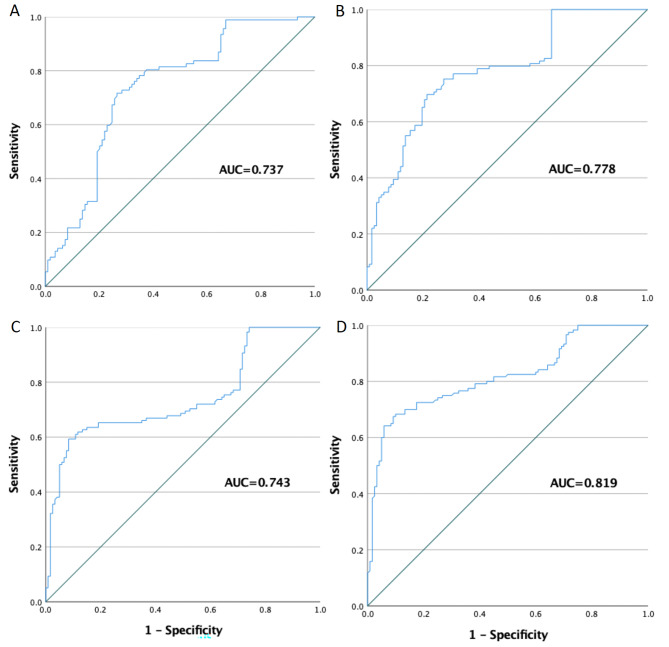
**ROC curve analysis using plasma anti-HBc levels for discriminating CHB patients that achieved HBsAg seroclearance and patients that remain HBsAg (+).** All four ROC curves reached statistical significance at p < 0.001. (A) Plasma anti-HBc level at 10–8 years before HBsAg seroclearance yielded an AUC (the areas under the ROC curve) of 0.737 [95% CI 0.668–0.806]. (B) Plasma anti-HBc level at 8–5 years before HBsAg seroclearance yielded an AUC of 0.778 [95% CI 0.718–0.838]. (C) Plasma anti-HBc level at 3–1 years before HBsAg seroclearance yielded an AUC of 0.743 [95% CI 0.678–0.807] (D) Plasma anti-HBc level at 1–0 years before HBsAg seroclearance yielded an AUC of 0.819 [95% CI 0.764–0.873]

The role of plasma anti-HBc in predicting subsequent HBsAg seroclearance was analyzed by calculating the AUCs at various time points. As shown in Fig. 3, plasma anti-HBc levels up to 10 years ago can be used to predict the incidence of HBsAg seroclearance. Plasma anti-HBc levels at 10–8 years before HBsAg seroclearance, at the cut-off value of 112.45 COI, produced an AUC of 0.737 with 72.8% sensitivity and 71.6% specificity for discriminating S-losers from controls. Plasma anti-HBc levels at 8–5 years before HBsAg seroclearance, at the cut-off value of 112.30, produced an AUC of 0.778 with 70.6% sensitivity and 76.1% specificity for discriminating S-losers from controls. Plasma anti-HBc levels at 5–3 years before HBsAg seroclearance yielded an AUC = 0.657 and was not considered as an acceptable predictive tool (ROC curve not shown). Plasma anti-HBc levels at 3–1 years before HBsAg seroclearance, at the cut-off value of 83.55, produced an AUC of 0.743 with 61.0% sensitivity and 89.2% specificity for discriminating S-losers from controls. Plasma anti-HBc levels at 1–0 years before HBsAg seroclearance, at the cut-off value of 82.50 COI, produced the highest AUC of 0.819 among all time points with 68.3% sensitivity and 90.0% specificity for discriminating s-losers from controls.

As qHBsAg was lower among patients who subsequently developed HBsAg seroclearance compared to those who remained persistently HBsAg+ (Table 1), we evaluated the role of combining qHBsAg and anti-HBc to predict subsequent HBsAg seroclearance. We categorized patients into qHBsAg ≥ 100 IU/mL or < 100 IU/mL – this cut-off was chosen because achieving this is a pre-requisite of the widely accepted alternative treatment endpoint of ‘partial cure’ in chronic hepatitis B infection [36]. At the time point of -1 to 0 years before HBsAg seroclearance, a total of 71% and 29% subjects had qHBsAg < 100 IU/mL and qHBsAg ≥ 100 IU/mL, respectively. Among those with qHBsAg < 100 IU/mL, 70.4% developed HBsAg seroclearance within 1 year. In the subgroup with qHBsAg < 100 IU/mL, 88.2% patients with anti-HBc < 82.50 COI could develop HBsAg seroclearance within 1 year, compared to 54.1% patients with anti-HBc ≥ 82.50 COI (p = 0.002). Among subjects with qHBsAg > 100 IU/mL, none of them achieved HBsAg seroclearance within 1 year.

## Discussion

HBsAg seroclearance is currently regarded as the optimal endpoint in CHB infection, and is a accompanied by, at least partial, recovery of the long-standing immune dysfunction [37]. The exact initiating events for HBsAg seroclearance are not well delineated but likely involve multiple pathways in the virological aspects and the host immune system. Our results showed that lower plasma anti-HBc is predictive of subsequent HBsAg seroclearance as early as 10 years before the occurrence of the event. CHB patients that achieve HBsAg seroclearance have significantly lower plasma anti-HBc levels at every observation time point prior to HBsAg seroclearance. As the ROC curve analysis showed, both plasma anti-HBc levels measured at a given time point or longitudinal plasma anti-HBc levels can be utilized as useful metrics to predict the occurrence of subsequent HBsAg seroclearance. Low plasma anti-HBc levels suggest a higher probability of clearing HBsAg and reaching the treatment-endpoint of CHB, implying a good prognosis in the foreseeable future, especially in patients with low HBV DNA levels. Combining with quantitative HBsAg < 100 IU/mL, a pre-requisite to be considered as ‘partial cure’, anti-HBc < 82.5 COI identified 88.2% patients who would develop HBsAg seroclearance within 1 year, compared to 54.1% who had anti-HBc ≥ 82.5 COI. With constant monitoring of plasma anti-HBc levels, which are significantly lower and continue to decline during the progression of the disease, anti-HBc can be used to evaluate the prognoses of CHB patients.

Many studies have investigated whether serum anti-HBc levels correlate with host and viral factors such as treatment response, fibrosis, seroconversion events [38–41], but only until recently was the correlation between serum anti-HBc and HBsAg seroclearance found by making use of cross-sectional or longitudinal anti-HBc data. The result of this study is consistent with the findings of studies that compare serum anti-HBc levels in CHB patients that developed HBsAg seroclearance and those who remained HBsAg seropositive. One study found that low anti-HBc level (< 3 log IU/mL) was associated with undetectable HBV DNA and HBsAg seroclearance in 10 years, especially patients with low HBsAg titres (< 10^2^ IU/mL), and reported a ROC curve with AUC of 0.82 for distinguishing HBeAg-negative patients who can achieve HBsAg seroclearance [33]. Another study found that low baseline anti-HBc IgG levels (< 11RLU), old age (≥ 50 years), and high ALT levels (≥ 40 IU/L) were associated with HBsAg seroclearance in NUC-treated HBeAg(-) patients [34]. To our knowledge, this is the first study that identifies a significant trend of decreasing plasma anti-HBc levels that preceded spontaneous HBsAg seroclearance in treatment-naïve HBeAg-negative CHB patients.

One of the classic traits specific to persistent HBV infection is that the hosts tend to have late, weak, narrowly focused cytotoxic T lymphocyte (CTL) responses that quickly show attenuation, in contrast to the vigorous, polyclonal, and multi-specific CTL responses in acutely infected patients who are able to achieve rapid viral control with rapid clearance of HBsAg from the blood, i.e. acute self-limiting HBV infection [42]. This suggests the key to viral clearance is generating immune responses that simultaneously target multiple viral antigens. Hepatitis B core antigen (HBcAg) is one of the HBV viral antigens recognized by the host immune system. Hosts seroconvert to anti-HBc as early as 3 weeks upon exposure [43]. HBcAg can trigger innate, humoral and cellular immune responses [44–46]. In particular, the T cell response to various epitopes of HBV including HBcAg is strong in acute self-limiting HBV infection, but is weak and quickly exhausted in chronic HBV infection [47, 48]. CTLs specific to all viral antigens do exist in the liver of CHB patients, but in very low levels. On the other hand, the antibody response to HBcAg comes off strong in chronically infected patients but have no direct antiviral effects [32]. It is generally reckoned that anti-HBc antibodies do not neutralize HBV and have little to no effect on viral clearance since they are present in high titers during the HBeAg-positive chronic infection phase, in which the host experiences a high degree of viremia and the immune system “tolerates” the virus. In addition, antiviral treatment and normalization of liver enzymes led to functional reduction in HBcAg-specific B cells and anti-HBc levels [46]. The role of the antibody response to HBcAg in the immunopathogenesis of HBV remains unclear [42, 49].

However, depending on phases, anti-HBc can either serve as a useful predictive factor for treatment outcome or a surrogate indicator that reflects HBV replication. Studies have shown higher anti-HBc levels were associated with higher rates of HBeAg seroconversion in HBeAg-positive patients [50]. Meanwhile, in HBeAg-negative patients, studies found that higher levels of anti-HBc were associated with relapse in HBeAg-negative patients and were disadvantageous for HBsAg seroclearance [51, 52]. The contrast may be explained by the difference in viral load between HBeAg-positive and HBeAg-negative patients. In HBeAg-positive patients, HBV titre is high and HBcAg is produced in excess, which cause hyper immune responses and anti-HBc production that favour HBeAg seroconversion. In HBeAg-negative patients, they have entered a prolonged period of viral quiescence with effective immune control in which anti-HBc level is a direct reflection of intrahepatic HBcAg load, which correlates with transcriptional activity of cccDNA in the liver [53]. In other words, a high anti-HBc level is an indication of high viral burden, and hence an unfavourable condition to HBsAg seroclearance, the hallmark which implies silencing of cccDNA. Anti-HBc’s non-neutralizing nature and its positive correlation to HBV DNA during the HBeAg-negative phase together can reasonably explain the result of this study, that low anti-HBc levels are favourable to HBsAg seroclearance and can serve as a predictive factor of this endpoint in the next 10 years.

Our study has two limitations. Firstly, the retrospective nature of the study precluded additional experiments to be conducted for providing mechanistic insights into how declining anti-HBc has contributed to HBsAg seroclearance. Secondly, we did not have plasma anti-HBc and anti-HBs data after achieving HBsAg seroclearance.

In conclusion, plasma anti-HBc level is negatively associated with HBsAg seroclearance and begins to decline 10 years prior to HBsAg seroclearance, which can serve as a potential predictor in predicting HBsAg seroclearance among patients with HBeAg-negative chronic hepatitis B infection. Combining two biomarkers i.e., qHBsAg and anti-HBc, is potentially useful to identify subjects who will develop subsequent HBsAg seroclearance.

## Data Availability

All data supporting the findings of this study are available within the paper.
